# Making drug delivery in the ICU safer: the implementation of advanced computerised intravenous infusion pumps

**DOI:** 10.1186/cc11108

**Published:** 2012-03-20

**Authors:** A Dimech, A Le Page, P Gruber, T Wigmore

**Affiliations:** 1 The Royal Marsden, London, UK

## Introduction

Drug administration errors account for approximately 78% of all medical errors occurring in ICUs [1,2]. The aim of this project was to introduce advanced computerised infusion pumps with in-built drug safety software, so-called smart pumps, in the ICU to facilitate safer drug administration.

## Methods

The working group consisted of an ICU pharmacist, clinical nurse specialist and consultant intensivist. A drug library was constructed by the ICU pharmacist and consultant intensivist and loaded onto the infusion pumps. The selection of drugs and dose limits were carefully considered to ensure that they were within the boundaries of normal usage so as not to impede patient management whilst maximising patient safety. Super users then provided individualised training to 85 ICU nurses. In the UK there have been 50 system implementations to date. The national average for compliance with the use of the software is 50%.

## Results

Feedback following training with the new system was very positive. In our ICU, utilisation of the drug safety software during drug administration was 94% within the first 6 months. Of 18,000 drug infusions, only 1,000 were used outside the drug safety software. There were seven over-rides from the high limit. Of these, two were for furosemide where there was a genuine clinical need for a higher dose. On two other occasions the software prevented insulin being administered at 30 units/hour and potassium at 100 mmol/hour. The number of drug errors reduced from three to zero during the study period. This demonstrated that the design of our package was sensitive enough to ensure safe drug administration and sufficiently practical to enable consistent use of the system.

## Conclusion

We have demonstrated that the introduction of an advanced computerised infusion pump system in the ICU can provide a safer drug administration environment if the appropriate health professionals are selected to implement the system, the drug library is constructed carefully and a comprehensive training package is applied.
